# Supplementary material for: Concurrent RB1 loss and BRCA-deficiency predicts enhanced immunological response and long-term survival in tubo-ovarian high-grade serous carcinoma
Source: medRxiv. 2023 Nov 10:2023.11.09.23298321. Preprint. [Version 1] doi: 10.1101/2023.11.09.23298321 (PMC10659507; doi:10.1101/2023.11.09.23298321)
Supplement: Supplement 14 [file NIHPP2023.11.09.23298321v1-supplement-14.pdf]

medRxiv preprint doi: https://doi.org/10.1101/2023.11.09.23298321; this version posted November 10, 2023. The copyright holder for this preprint (which was not certified by peer review) is the author/funder, who has granted medRxiv a license to display the preprint in perpetuity.
It is made available under a CC-BY 4.0 International license .

Supplementary Figure S1. Patients and tumour samples analysed in this study.

Number of patients included in each molecular analysis. HGSC, tubo-ovarian high-grade serous ovarian carcinoma; ENOC, endometrioid ovarian carcinoma; OS, overall survival.

Supplementary Figure S2. Combined p53 and RB1 protein expression in ENOC.

(A) Correlation between RB1 and p53 tumour expression in patients with endometrioid ovarian carcinoma (ENOC). Chi-square *P* value is reported. (B) Kaplan-Meier estimates of overall survival in patients with ENOC by combined RB1 and p53 tumour expression status.

Supplementary Figure S3. Drug sensitivity in HGSC cell lines with innate *RB1* and/or *BRCA1* alterations.

(A) Summary of the molecular features of innate HGSC cell models, including mutations in key genes (*TP53*, *CDKN2A*, *BRCA1*, *BRCA2*), copy number alterations in *CCNE1*, and protein expression of RB1 and p16. (B) IC50 of high grade serous ovarian cancer cell lines after treatment with cisplatin (72 hours), paclitaxel (72 hours), or olaparib (120 hours). ND, Not

medRxiv preprint doi: https://doi.org/10.1101/2023.11.09.23298321; this version posted November 10, 2023. The copyright holder for this preprint (which was not certified by peer review) is the author/funder, who has granted medRxiv a license to display the preprint in perpetuity.
It is made available under a CC-BY 4.0 International license .

determined. (C) Viability of high-grade serous ovarian cancer cell lines after treatment with cisplatin (72 hours), paclitaxel (72 hours), or olaparib (120 hours). Data are expressed as mean ($n = 3$ replicates) ± standard error of the mean (SEM). For some points, error bars are shorter than the height of the symbol and are not visible.

Supplementary Figure S4. Cell proliferation and cell cycle distribution of HGSC cell lines with *RB1* knockout.

(A) CRISPR/Cas9 knockout of *RB1* in 3 patient-derived ovarian cancer cell lines with different *BRCA1/2* and p16 backgrounds. The bar graph indicates *RB1* mRNA expression levels determined by RT-PCR ($n = 3$) in single-cell clones confirming *RB1* wildtype (WT) and knockout (KO) compared to heterozygous colonies without gene editing (Scramble). Representative Western Blots show p16 protein levels compared to GAPDH loading controls in each cell line and clone. Images of p16 IHC in AOCS parental cell lines are included confirming the respective p16 status. (B) Proliferative capacity of 3 patient-derived HGSC cell lines (*RB1* wild-type, WT and *RB1* knockout, KO clones) measured by IncuCyte Zoom live-cell imaging. Data represent mean ± SEM confluency after 20-25% starting confluency from three to six independent experiments. Dashed line denotes 75% confluency. (C) Cell cycle distribution following *RB1* CRISPR knockout. Proportion of cells in G0G1, S or G2/M phase 24 hours after treatment with DMF, cisplatin or paclitaxel at half the IC50 determined per cell line and drug, analysed by flow cytometry. Mean proportion ± SEM of three independently performed experiments are shown. Distribution was compared between *RB1* WT and KO clones using unpaired t test (ns, not significant; $^*P < 0.05$).

Supplementary Figure S5. Mutational signatures in homologous recombination deficiency and *RB1* subgroups.

medRxiv preprint doi: https://doi.org/10.1101/2023.11.09.23298321; this version posted November 10, 2023. The copyright holder for this preprint (which was not certified by peer review) is the author/funder, who has granted medRxiv a license to display the preprint in perpetuity.
It is made available under a CC-BY 4.0 International license .

Boxplots show the relative proportion (y-axis) of genome-wide mutational signatures[26] according to homologous recombination deficiency (HRD) and *RB1* status. Boxes show the interquartile range (25-75th percentiles), central lines indicate the median, dots represent each sample, whiskers show the smallest and largest values within 1.5 times the interquartile range, red triangles indicate the mean, and dotted lines join the mean of each subgroup to visualise the trend. The Kruskal–Wallis test *P* values displayed are Benjamini-Hochberg adjusted and the signatures are ordered by their significance. Pair-wise Mann-Whitney-Wilcoxon test adjusted *P* values are also reported. HRP, homologous recombination proficient.

Supplementary Figure S6. Genomic and clinical characteristics by combined homologous recombination deficiency and *RB1* status.

Boxplots show numerical clinical and genomic features (y-axis) according to homologous recombination deficiency (HRD) and *RB1* status. Boxes show the interquartile range (25-75th percentiles), central lines indicate the median, dots represent each sample, whiskers show the smallest and largest values within 1.5 times the interquartile range, red triangles indicate the mean, and dotted lines join the mean of each subgroup to visualise the trend. The Kruskal–Wallis test *P* values displayed are Benjamini-Hochberg adjusted and the features are ordered by their significance. Pair-wise Mann-Whitney-Wilcoxon test adjusted *P* values are also reported. Features include *BRCA1*- and *BRCA2*-type CHORD (Classifier of HOmologous Recombination Deficiency) scores; mean HRD scores (scarHRD); absolute numbers of structural variants (SVs), including deletions (DEL), duplications (DUP), intrachromosomal rearrangements (ITX), and inversions (INV); relative expression levels of *PCNA* and *MCM3*; proportion of whole-genome loss-of-heterozygosity (LOH); number of predicted neoantigens and variants per megabase (Mb); age of patients at diagnosis; progression-free and overall survival; cancer cell purity and ploidy; absolute CIBERSORTx scores; proportion of Ki-67

medRxiv preprint doi: https://doi.org/10.1101/2023.11.09.23298321; this version posted November 10, 2023. The copyright holder for this preprint (which was not certified by peer review) is the author/funder, who has granted medRxiv a license to display the preprint in perpetuity.
It is made available under a CC-BY 4.0 International license .

positive tumour cells were available for *n* = 59 primary tumours as previously measured by immunohistochemistry[7]. HRP, homologous recombination proficient.

Supplementary Figure S7. Gene alterations across *BRCA* and *RB1* altered subgroups.

Proportion of tumours with alterations in genes of interest for each subgroup. WT, wild-type; MUT, mutation; HRP, homologous recombination proficient. Genes are ordered by significance using Fisher's exact test; Benjamini-Hochberg adjusted *P* values are reported.

Supplementary Figure S8. Differentially expressed genes.

Bars indicate the number of differentially expressed genes (Benjamini-Hochberg adjusted *P* value < 0.05) between HGSC tumours grouped by HRD and/or *RB1* status as shown. Differential gene expression analysis was performed using DESeq2 to determine fold change of gene expression between groups (see Supplementary Table 7 for full DESeq2 results). HRP, homologous recombination proficient; HRD, homologous recombination deficient; RB1wt, *RB1* wild-type; RB1m, *RB1* altered.

<u>Supplementary Table captions:</u>

Supplementary Table S1.

Details of participating Ovarian Tumor Tissue Analysis (OTTA) consortium studies and ethics approval.

Supplementary Table S2.

Number of patients by study and histotype.

Supplementary Table S3.

Clinical characteristics of patients diagnosed with high-grade serous ovarian cancer.

Supplementary Table S4.

medRxiv preprint doi: https://doi.org/10.1101/2023.11.09.23298321; this version posted November 10, 2023. The copyright holder for this preprint (which was not certified by peer review) is the author/funder, who has granted medRxiv a license to display the preprint in perpetuity.
It is made available under a CC-BY 4.0 International license .

Clinical features of patients with endometrioid ovarian cancer

Supplementary Table S5.

Clinical characteristics of patients with high-grade serous ovarian cancer according to *BRCA* and RB1 status.

Supplementary Table S6.

Relative expression of *BRCA1* and *RB1* by qPCR in AOCS30 CRISPR knockout model.

Supplementary Table S7.

Differential gene expression analysis comparing transcriptomes of tumours based on *BRCA* and *RB1* alteration status.

Supplementary Table S8.

Summary of cell lines used in this study.

Supplementary Table S9.

Summary of gene alterations and expression found in cell lines.

Supplementary Table S10.

Sequence of single guide RNA used for CRISPR gene knockout.

Supplementary Table S11.

Antibodies and reagents used for this project.

Supplementary Table S12.

List of primer sequences used in the study.

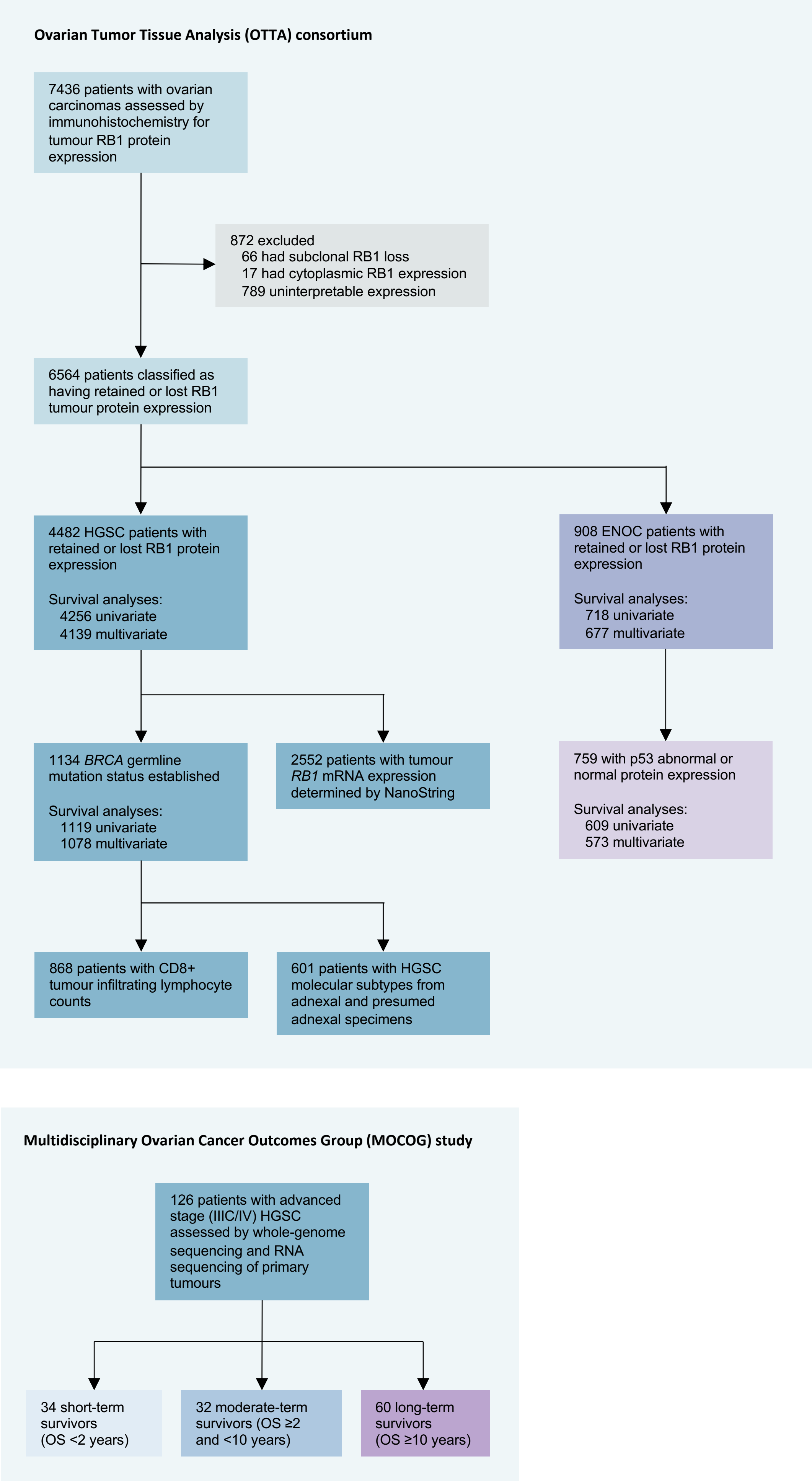

Supplementary Figure S1.

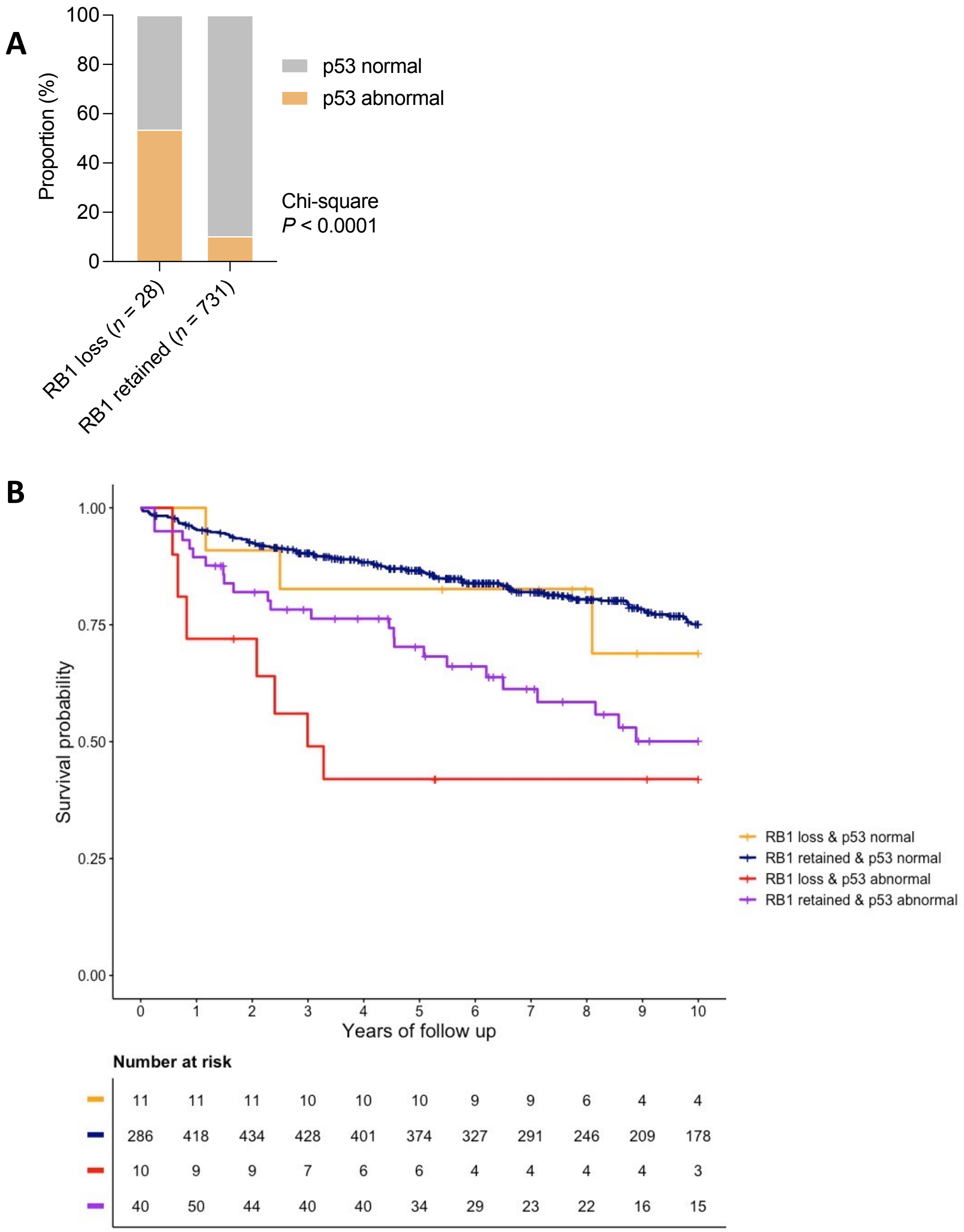

Supplementary Figure S2.

A

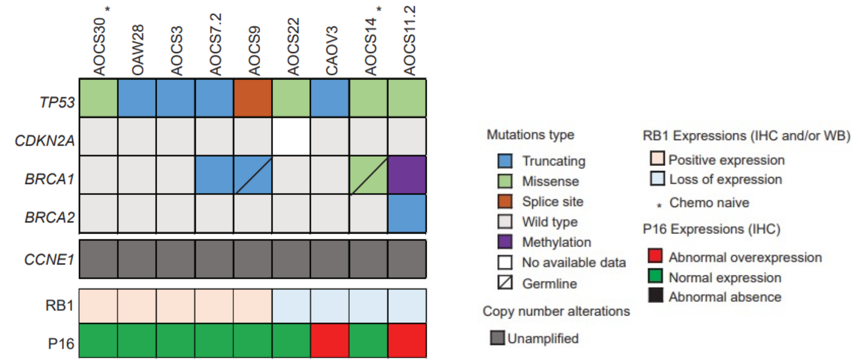

B

RB1/BRCA1	WT/WT			WT/gmut		Loss/WT		Loss/gmut	Loss/ methy
Cell line	AOCS30	OAW28	AOCS3	AOCS7.2	AOCS9	AOCS22	CAOV3	AOCS14	AOCS11.2
Cisplatin (μM)	0.5955	0.8069	27.95	0.2933	5.142	1.41	2.303	0.1747	0.705
Paclitaxel (nM)	0.6272	0.6259	ND	107.3	ND	0.6339	0.07528	0.4297	14.23
Olaparib (μM)	2.694	0.9433	36.68	6.654	21.19	8.33	19.86	0.7811	21.23

C

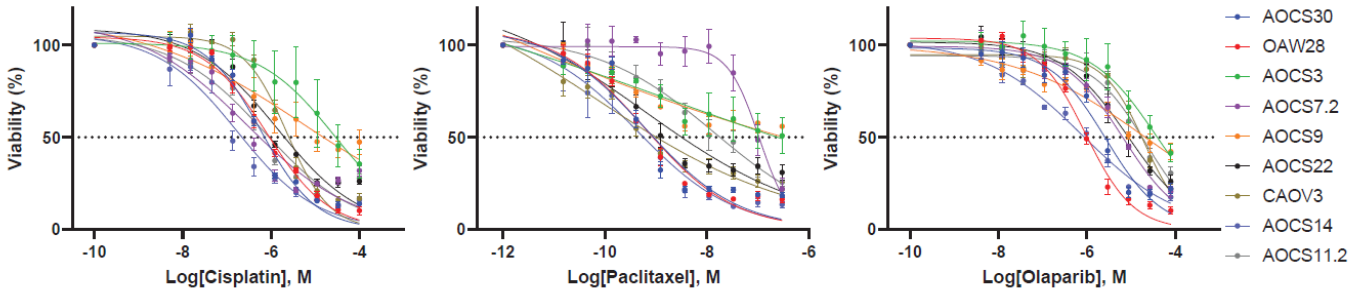

Supplementary Figure S3.

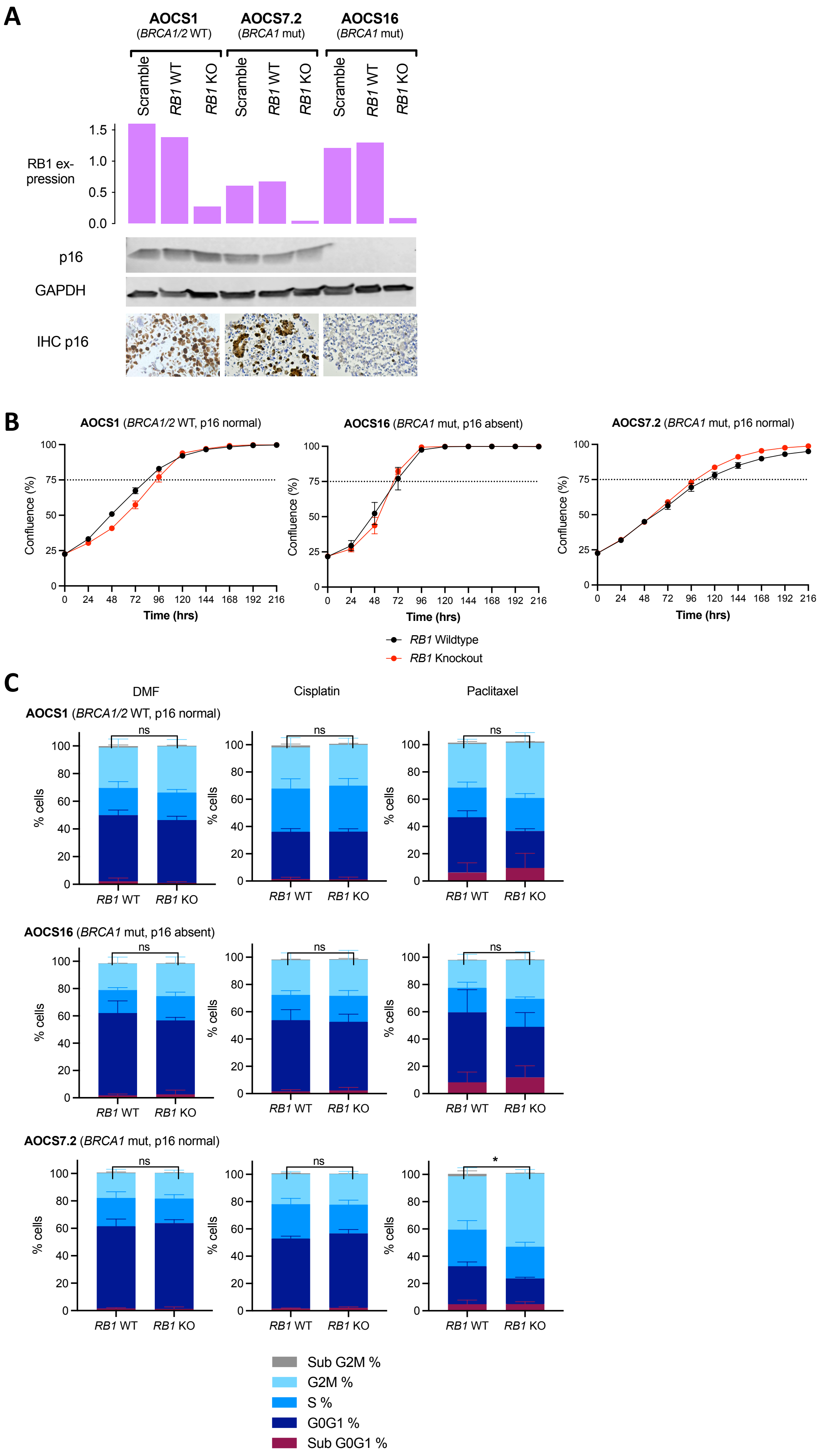

Supplementary Figure S4.

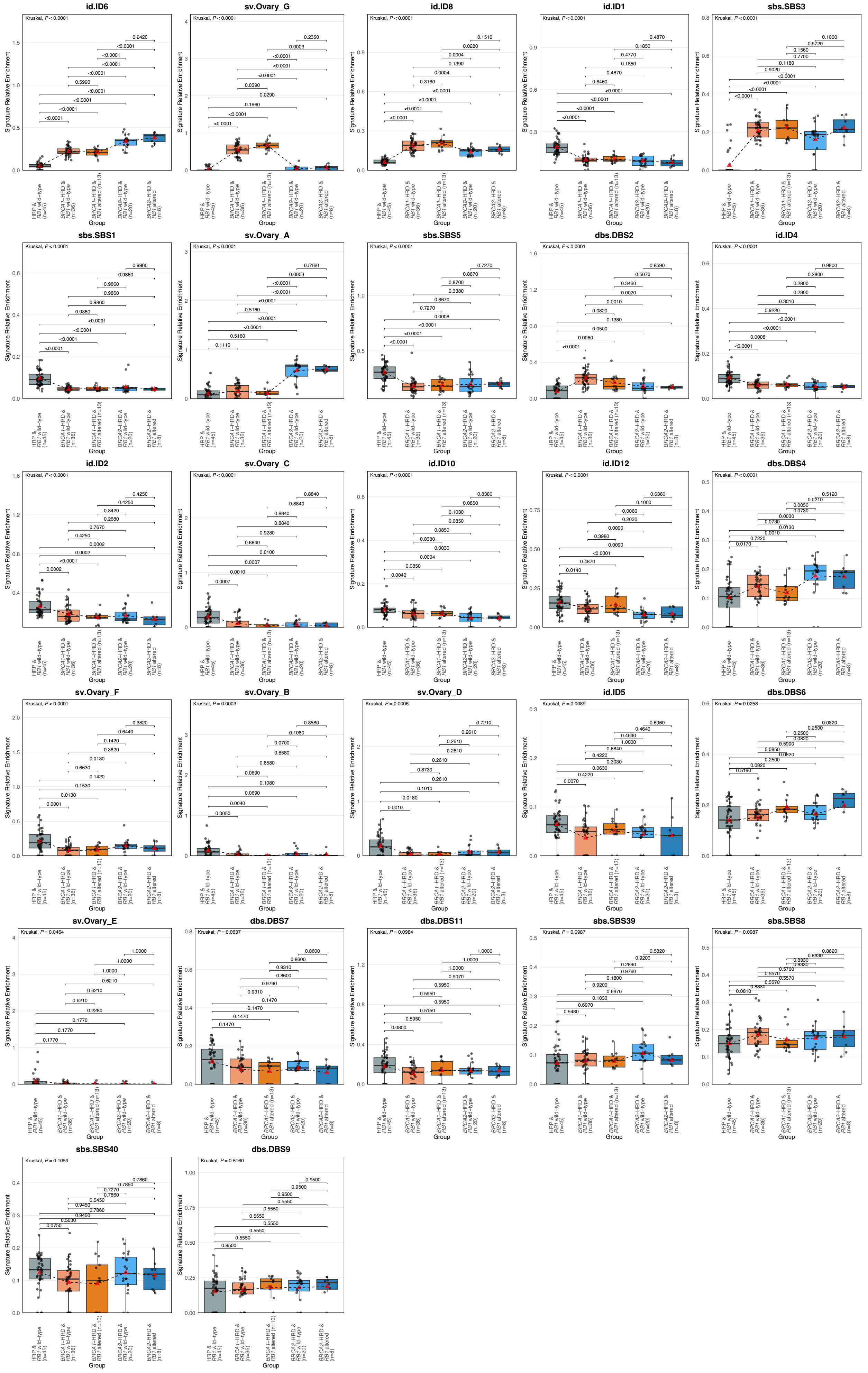

Supplementary Figure S5.

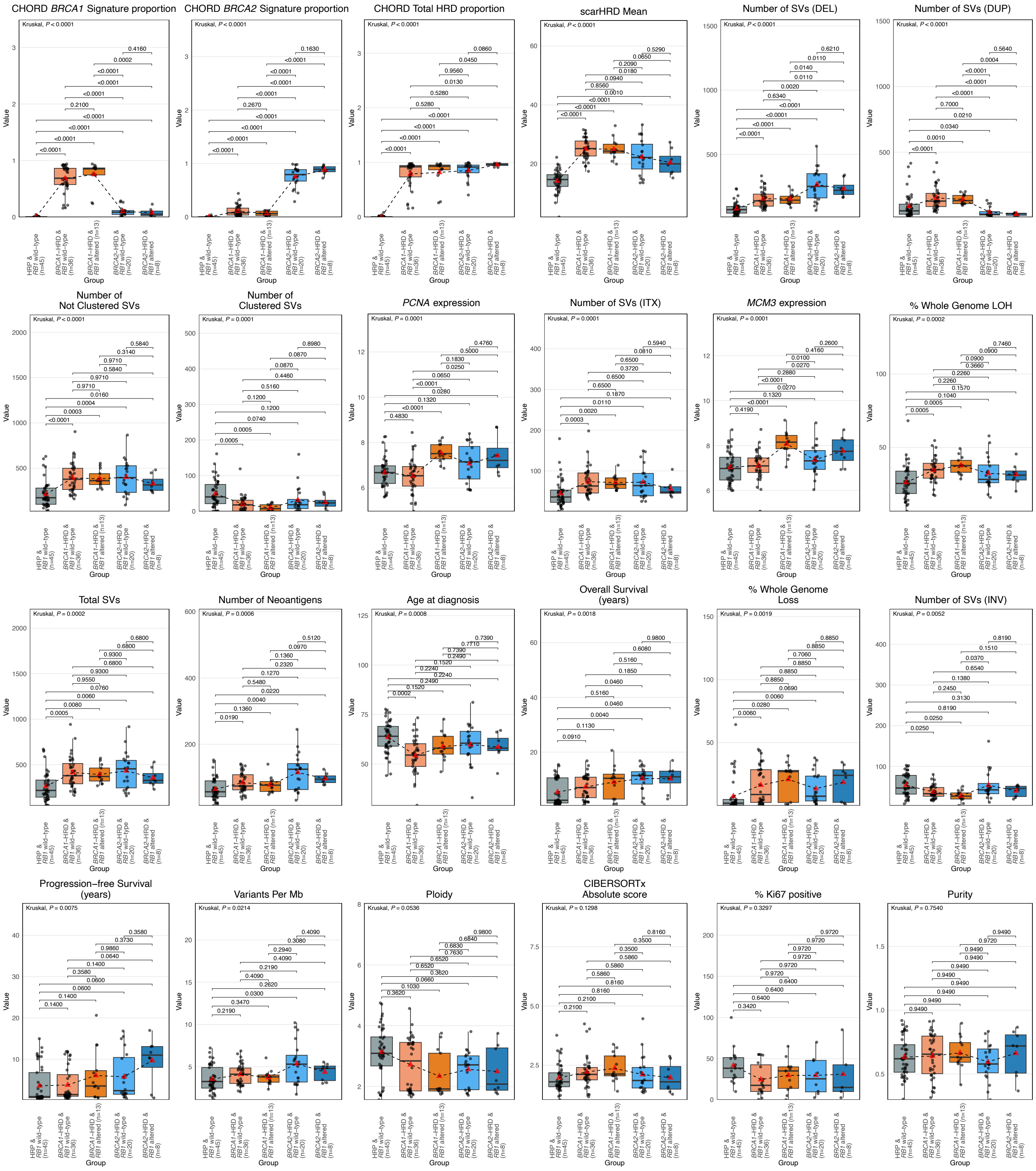

Supplementary Figure S6.

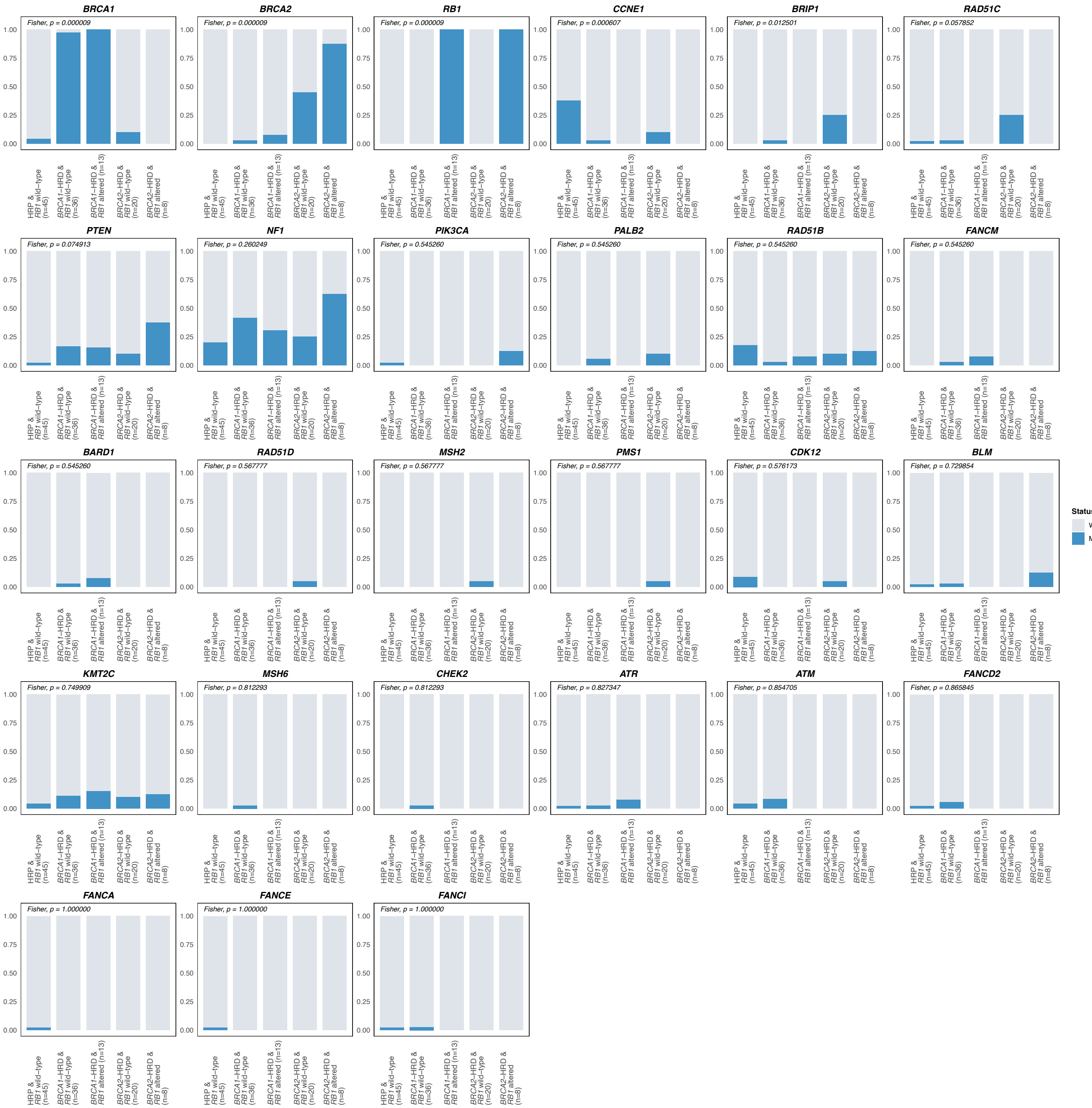

Supplementary Figure S7.

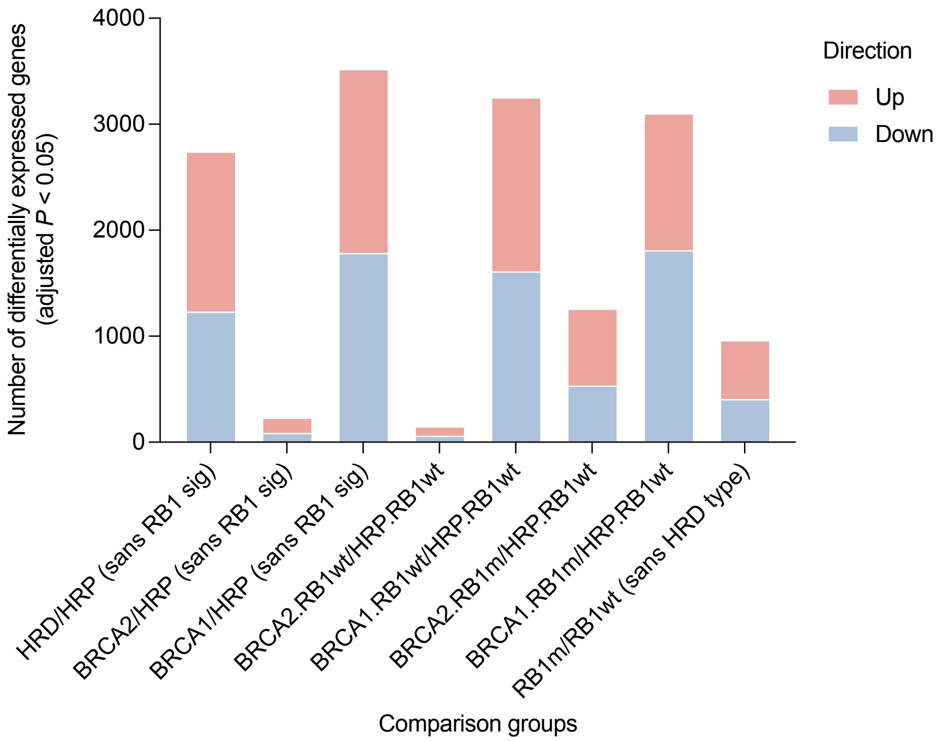

Supplementary Figure S8.
